# Voltage-Gated Sodium Channels: A Prominent Target of Marine Toxins

**DOI:** 10.3390/md19100562

**Published:** 2021-10-05

**Authors:** Rawan Mackieh, Rita Abou-Nader, Rim Wehbe, César Mattei, Christian Legros, Ziad Fajloun, Jean Marc Sabatier

**Affiliations:** 1 Faculty of Sciences 3, Department of Biology, Lebanese University, Campus Michel Slayman Ras Maska, Tripoli 1352, Lebanon; rawan97mck@gmail.com (R.M.); ritaabounaderr@hotmail.com (R.A.-N.); 2 Biology Department, Faculty of Arts and Sciences, American University of Beirut, Beirut 1107 2020, Lebanon; rgw00@mail.aub.edu; 3 University of Angers, INSERM U1083, CNRS UMR 6015, MITOVASC, SFR ICAT, 49000 Angers, France; cesar.mattei@univ-angers.fr; 4 Laboratory of Applied Biotechnology (LBA3B), Azm Center for Research in Biotechnology and Its Applications, Doctoral School of Sciences and Technology (EDST), Lebanese University, Tripoli 1300, Lebanon; 5 The Institute of NeuroPhysiopathology (INP), Aix-Marseille Université, CNRS, INP, 13385 Marseille, France

**Keywords:** VGSCs, marine toxins, neurotoxins, pufferfish, shellfish, sea anemone, cone snail

## Abstract

Voltage-gated sodium channels (VGSCs) are considered to be one of the most important ion channels given their remarkable physiological role. VGSCs constitute a family of large transmembrane proteins that allow transmission, generation, and propagation of action potentials. This occurs by conducting Na^+^ ions through the membrane, supporting cell excitability and communication signals in various systems. As a result, a wide range of coordination and physiological functions, from locomotion to cognition, can be accomplished. Drugs that target and alter the molecular mechanism of VGSCs’ function have highly contributed to the discovery and perception of the function and the structure of this channel. Among those drugs are various marine toxins produced by harmful microorganisms or venomous animals. These toxins have played a key role in understanding the mode of action of VGSCs and in mapping their various allosteric binding sites. Furthermore, marine toxins appear to be an emerging source of therapeutic tools that can relieve pain or treat VGSC-related human channelopathies. Several studies documented the effect of marine toxins on VGSCs as well as their pharmaceutical applications, but none of them underlined the principal marine toxins and their effect on VGSCs. Therefore, this review aims to highlight the neurotoxins produced by marine animals such as pufferfish, shellfish, sea anemone, and cone snail that are active on VGSCs and discuss their pharmaceutical values.

## 1. Introduction

Voltage-gated sodium channels (VGSCs) have been the topic of significant research and discussion for a considerable amount of time given their unique functions in electrical cell signaling. In 1952, Hodgkin and Huxley were the first to establish the existence of VGSCs and their crucial role in the generation of action potentials (APs) for which they won the Nobel Prize in 1963 [1,2]. VSGCs are activated by membrane depolarization, resulting in a fast and temporary Na^+^ influx before an intracellular loop quickly closes the pore [3]. These channels are very important for homeostasis, thus some genetic abnormalities in VGSC genes can result in a varied range of disorders known as "channelopathies" [4] such as muscle, cardiac, and neurological disorders [5]. Moreover, because of their ability to bind local anesthetics, VGSCs are the major target to treat different types of pain [6]. These ion channels are pharmacologically validated molecular targets for a large panel of clinically used drugs, such as antiarrhythmics, anticonvulsants, anesthetics, and analgesics [7]. Researchers have studied many pharmacological agents, including marine neurotoxins, to develop treatments for VGSC-related disorders and pain-relieving drugs. Neurotoxins produced by marine animals or microorganisms can bind to VGSCs at different sites, resulting in either activation or inhibition of the channel [2]. These toxins such as tetrodotoxin (TTX) and saxitoxin (STX), two pore-blocking toxins, have significantly improved our comprehension of the VGSCs function and structure [8]. Hence, this review aims to provide an overview of the main toxins produced by venomous marine animals and microorganisms that act on VGSCs and examine their pharmaceutical potential in the management of pain and some diseases. 

## 2. Voltage-Gated Sodium Channel (VGSC) Structure

**Figure 1:**
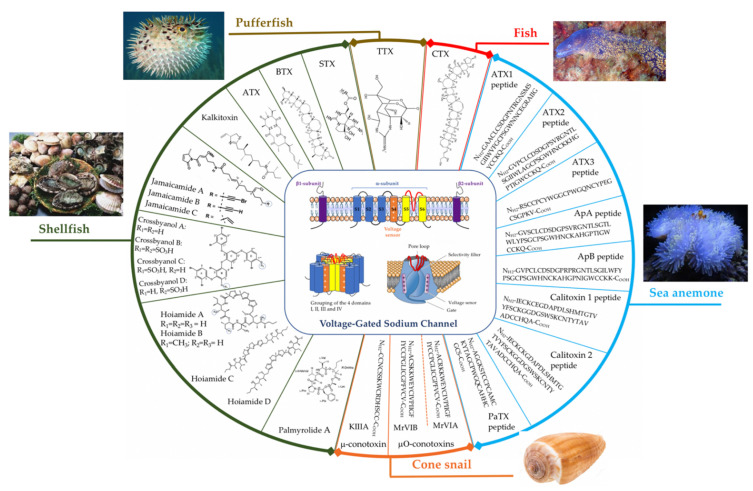
In the middle: overview of the VGSC structure including the different subunits: α, β1, and β2 (only one domain of the polypeptide is shown, in the top), as well as the selective pore formed by these subunits (at the bottom), and the main toxins present in marine animals that are active on these channels, as well as their identified chemical structures (peptide and non-peptide).

VGSCs are important members of the voltage-gated ion channel superfamily. These channels are known to transport ions between extra- and intracellular compartments in a voltage-dependent manner [9]. The initiation and propagation of APs in excitable cells are due to the presence of VGSCs, which allows Na^+^ influx. They also play an important role in non-excitable cells, such as T-lymphocytes, macrophages, endothelial cells, and astrocytes including Na(+)/K(+)-ATPase activity, as well as in regulation of effector functions such as phagocytosis metastatic activity and motility [10]. VGSC is a large protein constituted of two or three subunits: α (260 kDa) pore-forming subunit, which is linked to one or two β1–4 auxiliary subunits (30–40 kDa) [11]. The α subunit by itself is enough for the functional expression of the channel. However, the auxiliary β subunits are needed for the localization of the channel as well as its interaction with cell adhesion molecules, intracellular cytoskeleton, and extracellular matrix. β subunits are also crucial for the modification of the kinetics and voltage dependency of the channel’s gating [12]. α subunits contain four homologous, but not identical, domains (I–IV) in tandem. Each domain has six transmembrane helixes (S1–S6) and a pore-forming loop between the S5 and S6 segments [12,13] (Figure 1). Every third position in the S4 segments of each domain contains positively charged amino acid residues. These residues operate as gating charges, moving across the membrane to activate channels in response to membrane depolarization [12]. Subsequently, Na^+^ ions move under the influence of the transmembrane electrochemical gradient, leading to membrane depolarization, throughout the expanding phase of an action potential [13]. Regarding the inactivation gate, a small intracellular loop connecting the two homologous domains III and IV that folds into the channel structure is responsible for the blockage of the pore from the inside during sustained membrane depolarization [12]. Thus far, nine mammalian subunit isoforms have been discovered, resulting in nine VGSC subtypes (NaV1.1–NaV1.9) encoded by distinct genes [7]. Furthermore, the α subunit provides a binding site for a wide range of drugs such as antiarrhythmic, anti-epileptic, and local anesthetics, as well as various neurotoxins that target VGSC and can significantly change the channel’s activity [14].

## 3. Toxins from Marin Animals That Target VGSCs

### 3.1. From Fish

#### 3.1.1. Tetrodotoxin (TTX)

The neurotoxin, tetrodotoxin (TTX), is found in marine species, particularly in pufferfish. Marine species do not produce TTX by themselves, they rely on the presence of toxin-synthetizing bacteria (i.e., *Pseudoalteromonas*, *Pseudomonas*, *Vibrio*, *Aeromonas, Actinomycetes, Microbacterium, Serratia,* etc.) to produce the neurotoxin. These bacteria are either part of the animal’s microbiome or can bioaccumulate through the food chain [15]. The chemical formula of TTX is C_11_H_17_N_3_O_8_, and it contains a guanidinium group. TTX is water-soluble and stable in strong acidic solutions only [16]. It is highly toxic on the animal’s physiology. It suppresses almost any function that is dependent on the nerve and muscle, as well as the cell’s excitability. TTX has been used for years to study and identify the structure and function of VGSCs. Additionally, TTX exclusively blocks these channels when applied to the neuronal membrane’s exterior surface by binding to the toxin’s site 1 of α-subunit. However, when applied intracellularly, even at concentrations as high as 1 µM, it has no effects on the channel [16,17]. Also, Chen et al. have recently elucidated the mechanism of blocking VGSCs by TTX. They found that TTX blocks NaV1.4 via creating a network of hydrogen bonds (H-bonds) with the outer charged ring. Furthermore, the guanidinium group of TTX maintains a lateral orientation relative to the filter on blocking NaV1.4. The acidic residues on the outer membrane are essential for the stabilization of the H-bonds [18]. The guanidine groups in TTX are positively charged, while the residues, Glu755 and Asp400 in domain II and I of the channel, are negatively charged, resulting in the creation of a strong interaction. In addition, the guanidinium group is a relatively large molecule, making it difficult for TTX to penetrate through the channel. Consequently, the Na^+^ ion route becomes clogged and causes the blocking of the channel that inhibits the transmission of Aps impulses and results in loss of excitability and paralysis [8,19,20,21,22]. 

VGSCs are separated into two classes depending on their sensitivity to TTX: TTX-resistant (TTX-R), which needs concentrations in the micromolar range to efficiently be blocked by TTX, and TTX-sensitive (TTX-S), for which only nanomolars of TTX are sufficient to block them. Among the known TTX-S VGSCs, there are NaV1.1, NaV1.2, NaV1.3, NaV1.4, NaV1.6, and NaV1.7 subtypes. NaV1.5, NaV1.8, and NaV1.9 subtypes are TTX-R VGSCs (12). This selectivity of action makes TTX an important pain blocker agent; NaV 1.5, which is mainly expressed in the heart, is grouped under TTX-R class. 

**Figure 2:**
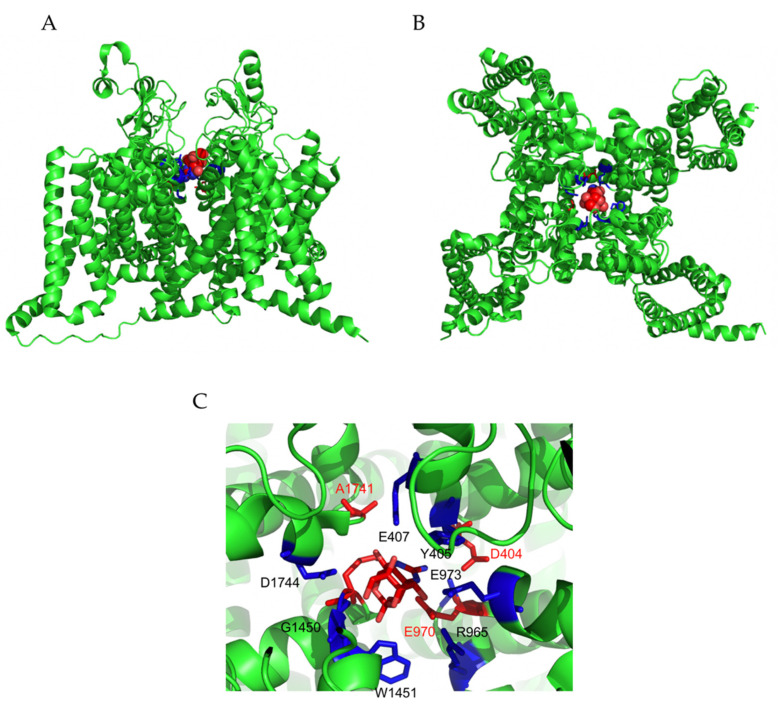
Interaction of TTX with Nav1.7 channel. Docking model of TTX-Nav1.7 channel interaction resolved by CryoEM (pdb code 6J8I, [31]). Nav1.7 channel is displayed as a cartoon, while TTX is shown as spheres. (**A**) Profile view; (**B**) top view. (**C**) Close-up showing the TTX-binding site within the pore. Lateral chains of amino acids interacting with TTX are labeled. In red are the amino acids that form the selective filter (DEKA). TTX is shown in the center of the pore in stick. The figures were prepared with PyMOL (DeLano W.L. (2010) The PyMOL Molecular Graphics System, version 1.6, Schrodinger, LLC, New York, NY, USA).

Given the critical role of VGSCs in pain signaling, TTX has become prominent as a therapeutic candidate for pain [23]. Several studies have verified the effect of TTX in inflammatory pain. When carrageenan, a pro-inflammatory drug, was administrated with TTX, mechanical and thermal hyperalgesia were reduced throughout the inflammatory response in rats [24]. Another study showed that TTX reduced thermal hypersensitivity in rats suffering from chronic inflammatory pain. Moreover, in the same study, when the inhibitory effect of TTX was compared with carbamazepine, an inhibitor of the voltage-gated sodium channel, it was found that the effect of TTX was 150 times greater than carbamazepine [25]. Earlier studies have demonstrated that, apart from inflammatory pain, TTX exerts an effect on acute pain [26,27,28]. Moreover, Marcil et al. have shown that TTX reduces visceral and neuropathic pain in Wistar rats, with no documented side effects to date, whereas morphine was shown to cause heavy sedation [29]. The previously mentioned results are compatible with the results of González-Cano et al., who demonstrated that blocking TTX-S sodium channels, but not NaV 1.7 alone (Figure 2), by TTX could be an effective treatment for visceral pain in viscero-specific mouse models [30].

TTX has been proven to be most efficient in neuropathic pains, particularly against cancer-related pain with tolerable toxicity and long-term safety [32,33]. Clinical studies have demonstrated that subcutaneous or intramuscular local injection of TTX is beneficial to treat cancer pain [32,34]. According to a recent study, in chemotherapy-induced neuropathic pain patients, 30 g BID of TTX given for 4 days every 3 weeks produced analgesia [35]. Moreover, when compared to TTX intramuscular injection, TTX pellets significantly improved safety in rats suffering from postherpetic neuralgia [36]. Furthermore, TTX-treated paclitaxel induced neuropathic pain in mice by reducing mechanical cold allodynia, and the development of thermal hyperalgesia [37]. The effect of TTX on burn-related pain has also been tested. Normally, opioids are used to treat the pain caused by burn injuries. However, due to the opioids’ negative side effects [38], TTX is considered a suitable alternative. Salas et al. suggested that systemic TTX could be used as a valuable rapid acting analgesic for burn injuries and could replace or reduce the use of opioid analgesics [39]. In addition, TTX has the potential to reduce thermal hyperalgesia and mechanical allodynia [39,40]. Pain-induced neurogenesis was greatly decreased when TTX was delivered locally and persistently to the sciatic nerve trunk [41]. Moreover, it has been demonstrated that a small dose of TTX reduces cue-induced increases in heroin and the associated anxiety [42]. 

Concerning the safety of TTX, a recent study examined the therapeutic dosages of TTX that could be administrated. The findings showed that all doses have an acceptable tolerability and PK profiles. Additionally, all doses demonstrated an absence of any clinically significant toxicity. Up to 45 μg of TTX was found safe and tolerated in terms of cardiac safety [43]. Like any other toxin, TTX may present some limitations such as the low efficacy of diffusion through diverse tissue barriers to the site of action, which means that the blockage of NaV will also be limited [44]. To surpass these difficulties and accomplish adequate amounts and duration of nerve fibers to create a blocking effect, high concentrations of the toxin are needed. This can result in considerable systemic toxicity. To improve the penetration and the analgesic signal, many studies have worked on coupling TTX with other compounds called chemical permeation enhancers reviewed before [45,46]. Moreover, epinephrine and bupivacaine have been previously used to improve the efficacy of TTX and prolong the analgesic effect [47,48]. Moreover, Santamaria et al. have proved that the negative side effects linked to the toxicity of TTX were remarkably reduced with epinephrine and yielded even greater results than with chemical permeation enhancers (CPEs), which are known to improve nerve blockage caused by site 1 sodium channel blockers such as TTX [49]. Finally, when TTX was combined with capsaicin, a component found in chili pepper that can cause sensory-selective peripheral nerve blockage, prolonged duration of local anesthesia was detected [50].

#### 3.1.2. Ciguatoxins (CTXs)

CTXs are lipophilic cyclic polyethers with more than 20 analogues that have been described thus far. They are synthesized by benthic dinoflagellates of the genius *Gambierdiscus* [51]. These toxins can contaminate the food chain from the dead coral reef, which is colonized by dinoflagellates, consumed by herbivorous fish that are themselves the prey of carnivorous fish. Fish carrying CTXs can store these toxins without being affected by their harmful effects. CTXs are heat-resistant and do not alter the organoleptic properties of the contaminated fish. In humans, CTXs are responsible for a complex human food syndrome called ciguatera, characterized by peripheral neurological symptoms (myalgias, cold allodynia, arthralgias, paresthesia, pruritus) and central symptoms (ataxia, headache) [52,53]. Cardiovascular signs—bradycardia and low blood pressure—and gastrointestinal signs—abdominal pain, vomiting, nausea—are also observed. All these symptoms persist for several days, and in most cases disappear spontaneously. There is no specific treatment for ciguatera, and the treatment is purely symptomatic [53]. The symptoms observed are like those described in neurotoxic shellfish poisoning (see Section 3.2.2.)

CTXs act like brevetoxins (BTXs) by binding to site 5 of NaV channels at the intracellular segments 6 and 5 of domains I and IV, respectively [54,55]. Their mode of action is therefore similar: they slow down the inactivation of the Na^+^ current, which facilitates the persistent entry of Na^+^ into the cells. They also shift the activation of the Na^+^ current towards more negative potentials [56,57]. These toxins are therefore activators of NaV channels. The result is the generation of repetitive action potentials, characteristic of the exacerbated excitability, which is at the origin of the symptoms observed in humans [58]. Ciguatera is a disease present in the Pacific Ocean, the Indian Ocean, and the Caribbean area.

### 3.2. From Shellfish

#### 3.2.1. Saxitoxin (STX)

STX is a non-peptide neurotoxin found in shellfish and produced by marine dinoflagellates that accumulate in shellfish via the food chain. Three species of dinoflagellate, *Alexandrium*, *Pyrodinium*, and *Gymnodinium*, are responsible for STX production in the marine environment [59]. STX is known as a “paralytic shellfish toxin” (PST) since it is responsible of a food intoxication called “paralytic shellfish poisoning” (PSP) syndrome [8,60]. The molecular formula of STX is C_10_H_17_N_7_O_4_. It is considered as one of the most lethal toxins for humans. Poisoned persons can develop symptoms within 30 minutes beginning with a burning or tingling sensation on the lips and face and progressing to total numbness that may expand to the fingers and toes and reach the extremities. Other symptoms may include perspiration, vomiting, diarrhea, and stomach cramps. An overdose (toxic dose in humans is 1–4 mg/person) of STX can cause death due to respiratory failure and cardiovascular shock [61,62]. To date, no specific antidotes to STX have been approved. Activated charcoal that remove unabsorbed poisons and artificial respiration are the two frequent treatments used for PSP in its early stages [61].

Aside from TTX, STX is a powerful neurotoxin that preferentially targets VGSCs. The mechanism of binding of STX on VGSCs occurs via binding to the pore-forming region of the alpha-loop VGSC, located between S5 and S6 (Figure 1). This mechanism is similar to that of TTX, as they both belong to guanidinium toxins [8,63]. TTX and STX were the primary neurotoxins discovered that bind at site 1 of VGSC [2]. The guanidinium groups confer a positive charge at physiological pH that will enable the toxin to bind VGSC at site 1. Later, the conductance of Na^+^ through the channel is blocked. STX interferes with the genesis of APs in neurons and skeletal muscle at nanomolar concentrations [2,8,59]. 

The systematic toxicity caused by STX constitutes a major obstacle for its clinical use as an anesthetic and analgesic agent. In addition, and unlike TTX, STX has been shown to cross the blood–brain barrier. Several studies have worked on developing methods that can reduce STX’s toxicity. STX was tested for its effect as an anesthetic agent in rabbit cornea, rat, and dog [64]. When given at high concentration, long term blockage is obtained but a systemic toxicity occurs. To decrease this toxicity, STX was combined with a vasoconstrictor agent. Researchers found that the systemic toxicity was reduced while the frequency of adequate blocks and their mean duration were enhanced [64]. Another study has demonstrated that STX acts as an efficient, safe, and long-term corneal anesthetic in rabbits after mechanical corneal abrasion and photorefractive keratectomy [65]. Site 1 blockers are found to prolong nerve blockade. Studies showed that the combination of tricyclic antidepressants, known for their role as local anesthetics, with STX prolonged the duration of local anesthesia [66]. Similarly, the co-administration of STX with dexmedetomidine, an α2-adrenergic receptor agonist, has expanded the analgesic effect in cornea without causing retardation in corneal wound healing. As a result, such combination can be effective in managing acute surgical and nonsurgical corneal pain [67]. Researchers have developed liposomal formulations containing a mixture of STX, bupivacaine, a local anesthetic that blocks the α subunit of VGSC, and dexamethasone as an inflammation reliever. The formed mixture has provided long-lasting local anesthetic effect with low systemic toxicity and caused sciatic nerve blockade in male Sprague–Dawley rats that lasted for up to 7.5 days. Systematic toxicity was only observed with high doses of dexamethasone that has boosted the release of liposomal STX. Formulations that only contain bupivacaine showed mild myotoxicity. Thus, regulated release of STX can provide long-term nerve blockade with low systemic and local toxicity [68]. 

STX possesses more than 50 natural analogues that block NaV in the same way that STX does, such as neosaxitoxin (neoSTX), decarbamoylsaxitoxin (dcSTX), and gonyautoxins I–III [69,70]. One of these analogues, ST-2530, was evaluated against human NaV1.7, which is involved in pain transmission. Results showed that pharmacologic blockage of NaV1.7 by a small-sized molecule agent with affinity for the channel’s resting state is sufficient for the production of an analgesic effect in a variety of preclinical pain models [70]. NeoSTX presents a low risk of side effects and might be used as a long-term local anesthetic [71,72,73,74]. Moreover, NeoSTX has been tested for its analgesic effect in piglet castration and was proven to be an effective and safe pain reliever [75]. Moreover, it has been demonstrated that NeoSTX is a long-acting local pain blocker used to treat patients suffering from bladder pain syndrome. NeoSTX exerts an analgesic effect in patients going through laparoscopic cholecystectomy [76,77]. Moreover, NeoSTX is considered to be a beneficial tool that reversibly inactivates various brain regions for an extended duration of time with low diffusion and without being harmful to neurons. Furthermore, NeoSTX can be suitable as a VGSC inhibitor in a variety of in vivo studies as well as for prospective therapeutic applications [78]. Gonyautoxin, one of STX analogs, has the potential to safely inhibit neural transmission of pain during knee arthroplasty and chronic tension-type headache [79,80].

#### 3.2.2. Brevetoxins (BTXs)

BTXs are lipid soluble neurotoxins found in shellfish and produced essentially by the dinoflagellate *Kerenia brevis.* BTXs can also be produced by other species such as *K. selliformis*, *K. papilionacea*, *K. mikimotoi*, *K. brevisulcata*, *Chatonella antiqua*, *Fibrocapsa japonica*, and *Heterosigma akashiwo* [81,82]. BTXs are a type of polyether neurotoxin with a ladder frame structure and two different types of backbone structures. Type 1: BTX-2, 3, 5, 6, 8, and 9 (brevetoxin B backbone) and type 2: BTX-1, 7, and 10 (brevetoxin A backbone). Notably, all BTXs can be considered as derivates of the BTX-1 and BTX-2 [2]. The most common symptoms of severe poisoning with BTXs are headache, gastroenteritis, diarrhea, nausea, sensory problems, cranial nerve dysfunction, scorching sensation in the rectum, bradycardia, ataxia, paresthesia, and ataxia. BTXs can also lead to a condition known as respiratory irritation syndrome [82,83,84,85]. BTX has been shown to bind to VGSCs, particularly to TTX-S VGSCs, interacting with α-subunit at site 5. This results in the inhibition of inactivation of VGSC while also shifting activation to more negative membrane potentials, causing conformational variation that leads to atypical channel opening and Na^+^ current increase [81,86,87,88]. Other studies identified S6 of DI and S5 of DIV to be involved in the development of neurotoxic receptor 5, using a photoreactive BTX-3 derivate as a probe [89]. BTXs are the only VGSC modifying toxins known to have the ability to stabilize multiple conductance levels [2]. A recent study has demonstrated that BTX-2 improved synapse density and dendritic arborization and of cortical layer V pyramidal neurons in the peri-infarct cortex in mice after a photothrombotic stroke. In addition, BTX-2 resulted in a significant amelioration in motor recovery [90].

#### 3.2.3. Antillatoxin (ATX)

ATX is a cyclic lipopeptide produced from pantropical marine cyanobacterium *Lyngbya majuscule* [91]. A cutaneous exposure to *L. majuscula* can cause dermatitis with burning sensations. ATX possesses neurotoxic effects in initial cultures of rat cerebellar granule cells, with thinning of neuritis, swelling of neuronal somata, blebbing of neurite membranes, respiratory irritation, and eye inflammation [92,93]. Co-administration of ATX with noncompetitive antagonists of the N-methyl-d-aspartate (NMDA) receptor such as MK-801 and dextrorphan can prevent the ATX-induced neurotoxicity. Additionally, TTX inhibits ATX’s effects [94,95]. It has been demonstrated that the lipopeptide ATX acts as a VGSC activator and interacts with its α-subunit. However, the precise binding site of ATX is still unrecognized [2,96]. Cao et al. have demonstrated that in cells heterologously expressing rat NaV1.2, rat NaV1.4, or rat NaV1.5-subunits, ATX was able to enhance Na^+^ influx. The results proved that the effectiveness of ATX was unique and was not shared before by any VGSC-subunit activators acting at sites 2 and 5. These findings show that ATX is a VGSC activator with distinct pharmacological features. Deciphering ATX’s mechanism of action and molecular determinants might provide additional insights into VGSC gating processes [96]. In Table 1, the other, less-documented toxins present in shellfish that are also active on VGSCs are presented. 

### 3.3. Toxins from Sea Anemones

Sea anemones belong to the *Cnidaria phylum*. Cnidarians are known to have an uncommon morphological and genetic variety. In fact, all cnidarians have specialized cells, named nematocytes, that comprise a small stinging apparatus known as nematocysts involved in defense and prey capture. These nematocysts enclose a high venom complex produced by nematocytes [105,106,107]. The venom complex produced by sea anemones contains neurotoxins that target VGSCs [108,109]. An intoxication by this toxin complex causes paralysis, pain, necrosis, local itching, erythema, swelling, neurotoxicity, and cardiotoxicity [110]. The cardiotoxicity consists of arrhythmias, produced early after depolarization due to inadequate NaV channel inactivation, and systolic stop due to Ca^2+^ ion overloading in myocardial cells [111].

Historically, the first isolated groups of neurotoxins affecting VGSC are ATX I, ATX II, and ATX III isolated from *Anemonia viridis* and known today as Av1, Av2, and Av3, respectively. Av1 (46 amino acids) and Av2 (47 amino acids) share a high sequence similarity [112,113]. This is in contrast to Av3, which has only 27 amino acids and is not related to either Av1 or Av2 in terms of sequence [114] (Figure 1). However, Av3 has been classified in a new different group along with Ea1 (PaTX), a toxin mainly isolated from *Entacmaea actinostoloides,* since they both lack the fourth disulfide bridge [115,116]. Rp1, Rp2, Rp3, and Rp4 are other toxins isolated from another species called *Heteractis paumotensis* and classified as a new group aside from the first group.

Concerning the mechanism by which sea anemone toxins act on VGSCs, it has been demonstrated that they bind to site 3, particularly at the residues in the extracellular loop between S3 and S4 in DIV [2]. In general, a neurotoxin that binds to this site slows down the inactivation process of the channel. This inactivation can be decelerated or even inhibited entirely, and these effects are linked to a slight hyperpolarizing shift in the activation of the channel, since S4 of DIV play a role in the voltage-dependent coupling between activation and fast inactivation [2].

Thus far, many toxins derived from sea anemones have been discovered and categorized under the 3 types: type I sea anemone toxins (Av2-like toxins); type II sea anemone toxins (Rp3-like toxins); and type III, which includes Av3 and Ea1 [115,117,118,119]. Toxins that belong to type I are powerful modulators of NaV channels that bind to site 3 at domain IV of the channel [120]. ATX II sequence was the first determined toxin [114] and was isolated from *Anemonia sulcate*, while Anthopleurin A was isolated from *Anemonia xanthogrammica.* Both toxins have inhibited the maximum gating charge of VGSCs. Mutagenesis studies showed that this reduction was due to the inhibition of S4 of domain IV in site 3, indicating the main binding site of these toxins [121]. Moreover, Anthopleurins are considered as type I NaV toxins and are isolated from the genus *Anthopleura* and can be potent on cardiac NaV [122,123]. ATX III, which belongs to type III, has also been found to bind to site 3. However, ATX III is not related to any other site 3 toxins [124]. Six ATX III toxins have been found until now from three different species (*Anemonia viridis*, *Dofleinia armata* and *Entacmaea quadricolor*). All of these toxins inhibit the inactivation of Na^+^ current. Moreover, studies showed that various residues are present on the surface of these toxins and make a hydrophobic patch that could be part of the NaV channel binding surface [115]. ATX I and ATX III are highly active in insects and crustaceans but not in mice, whereas ATX II is active in both mice and crustaceans [124,125].

Calitoxin I (Δ-hormotoxin-Cpt1a) and II (Δ-hormotoxin-Cpt1b), 79 amino acid residues long, belong to the type IV toxins. They are isolated from *Calliactis parasitica* and present a long chain and three disulfide bridges in common with types I and II [109,126]. Furthermore, they have a similar outcome on VGSCs as types I–III toxins [109]. In Table 2, the other, less documented toxins from sea anemones that are also active on the VGSCs are represented. 

### 3.4. Toxins from Cone Snails

The genus *Conus*, from the Conidae family, is a group of predatory gastropod mollusk that includes more than 500 different species. Cone snails belonging to this genus usually live in tropical waters [152]. They are categorized into three groups depending on their alimentation habits: mollusk hunters (molluscivorous), worm hunters (vermivorous), and fish hunters (piscivorous) [153]. They use their venom as a weapon to defend themselves and capture their prey [154]. The biotoxin of marine cone snails is a combination of many peptides that may vary between species. The venom of a single species can contain different toxins depending on its predatory or defensive use [155]. 

Conotoxins are small peptides rich in disulfide bridges found in the venom of conical snails [156]. First, cone snails produce propeptides in the secretory cells of their tubular venom duct. Then, the proteases cleave the precursor protein generating active conotoxins. The cone snail has a specialized harpoon-shaped root tooth used to inject its venom into its prey [157]. These conotoxins have shown a high affinity for various membrane receptors, ion channels, and transporters in the nervous system of target preys and predators that gave them a great value in the pharmaceutical field. According to their pharmacological activities and their molecular targets, conotoxins are grouped into different families [158]. Among the different families of conotoxins, four of them target VGSCs: μ-, μO-, δ-, and ί-conotoxins. Each of these families interacts with VGSCs by binding to specific sites on the channel α subunit. To date, only μ- and μO-conotoxin families exhibit pain-relieving properties in animal models [159]. μ- and μO-conotoxins inhibit the VGSC current—this happens when μO-conotoxins bind to an external site to the pore that modifies the channel gating and closes the channel [160], while μ-conotoxins bind to the external vestibule of the channel and thus sterically and electrostatically block the conductive pathway of ions [159]. Unlike μ- and μO-conotoxins, δ- and ί-conotoxins stimulate VGSC activity. The δ-conotoxins interact with the hydrophobic surface residues of the S3/S4 linker of domain IV site 6. However, this interaction results in the prolongation of the opening of the channels that extends action potentials and activates a persistent neuronal discharge, eventually deferring or inhibiting rapid inactivation [157,161]. ί-Conotoxins activate VGSC without affecting the inactivation by shifting the voltage dependence of activation to more hyperpolarized potentials or by improving the amplitude of the TTX-S Na^+^ current in DRG neurons [162]. 

μO-conotoxins belong to the O-superfamily of conotoxins, and they are known to be moderately selective inhibitors of VGSC TTX-R currents in rat DRG neurons. These toxins are hydrophobic peptides made up of 28 to 32 amino acids. Each toxin contains three intramolecular disulfide bonds [163]. The site 4 receptor on the NaV channels is their target; they block the flow of Na^+^ by preventing the voltage sensor in domain-2 from activating and consequently the channel from opening [160]. To date, only two μO-conotoxins have been identified: MrVIA and MrVIB from the venom of the mollusk-hunting species *C. marmoreus* [164]. MrVIA and MrVIB have high sequence homology, and they only differ by two residues. They are formed by 31-residue peptides with three disulfide bridges. The significance of MrVIA and MrVIB is in their ability to block TTX-R Na^+^ currents in mammalian DRG neurons 10 times more than TTX-S Na^+^ currents [163,165]. As μO-conotoxins act as gating modifiers, MrVIA inhibits Na^+^ current in a voltage-dependent way with a reduction in affinity after depolarizing voltage steps [160]. The inhibition of TTX-R by MrVIB is more selective than that of TTX-S neuronal VGSCs and even selective between the different subtypes of VGSC TTX-R (100 times more selective for NaV1.8 than NaV1.9 in DRGs) [163]. Of the nine NaV subtypes, NaV1.8 is expressed in peripheral sensory neurons and is present in the majority of nociceptive neurons [166]. It contributes to the action potentials of the Na^+^ current in the pain pathway. Therefore, it constitutes an interesting analgesic target. 

After examination of the MrVIA binding site in the NaV1.4 channels and competition experiments with the scorpion toxin Ts1, it was identified that the C-terminal pore loop of DIII is required for the binding of MrVIA to NaV1.4. Alternatively, another study that used site-directed NaV1.4 mutagenesis showed that the DII domain is the main binding site of MrVIA while the DIII domain plays a less important role [160,167]. Studies have shown that the region between the S6 segment of DI and the outer loop of DII in NaV1.8 is responsible for the strong affinity of the μO-conotoxin family for NaV1.8 [168]. Comparable to MrVIA and MrVIB, a new MfVIA toxin from *Conus magnificus* has been identified [169]. MfVIA is a 32-residue hydrophobic peptide that has the highest sequence homology to MrVIB. However, what differs is the selectivity towards VGSC subtypes. MfVIA is three times more effective against NaV1.4 and five times less effective against NaV1.2 than MrVIA or MrVIB. It has the ability to inhibit NaV1.4 and NaV1.8 at low nanomolar concentrations, while significantly higher toxin concentrations are required to block all other VGSC subtypes [169]. μO-Conotoxin includes a single toxin that binds and blocks the channel that is consistent with MfVIA and inhibits all VGSC subtypes except NaV1.2 [169]. Two new μO-conotoxin LtVIC and LtVIIA were discovered after sequencing of *C. litteratus*. These two toxins inhibit VGSC channels in the same way that μO-conotoxins do but their selectivity to VGSC subtypes must be further studied [170]. 

μ-Conotoxins are relatively small sized peptides of 16 to 20 amino acids containing three disulfide bonds; they are rigid and highly basic. At least 12 different μ-conotoxins have been identified thus far, with 6 of them being present in the venom of fish and mollusc-hunting cone snails [171]. The first identified μ-conotoxin was GIIIA from *C. geographus*. μ-Conotoxins can bind to the pore region of VGSCs. The selectivity of these toxins for VGSC subtypes was primarily due to the differences in the turret region (S5-P loop link), as the near-pore differences are minimal [172]. GIIIA is a selective and potent blocker for skeletal muscle as it inhibits NaV1.4 with a nanomolar concentration [173]. While PIIIA was the first to inhibit neuronal VGSC, studies have shown its capacity to inhibit skeletal VGSC in mammalians [174]. KIIIA and SIIIA are two small sized μ-conotoxins with high affinity, making them strong prospective therapeutic agents. Contrary to GIIIA, KIIIA binds to neuronal channel NaV1.2 and to NaV1.7 [175]. To understand how and to which VGSC domains do μ-conotoxins bind to, researchers have studied chimeras of rat and human NaV1.4 and NaV1.5. SIIIA and CnIIIC are potent blockers of the VGSC subtypes NaV1.2 and NaV1.4, but inactive at NaV1.8 and NaV1.5, alongside its inability to block NaV1.8 [176]. Furthermore, changes in the amino acid residue affect the affinity of μ-conotoxins to bind to the NaV channels [177]. 

Conotoxins have been shown to be promising candidates in the therapeutic field due to their selectivity and affinity to VGSC and additional membrane receptors and ion channels. An analogue of the χ-MrIA conotoxin is currently in phase II clinical trials for the treatment of neuropathic pain. Indeed, it inhibits the transporter of norepinephrine in a non-competitive way [178]. Preclinical trials have been completed for ω-CVID, but this toxin exhibited cytotoxic effects in phase II. For the management of pain and intractable epilepsy, human clinical trials are underway for contulakin-G and conantokine-G [179,180]. As a result, many conopeptides are being developed for the control of pain and various diseases such as Parkinson’s [181]. Furthermore, MrVIA, MrVIB, and MfVIA demonstrated high analgesic properties since they can inhibit NaV1.8, which has a fundamental role in the pathophysiology of pain. Nevertheless, the chemical composition of conopeptides as well as their folding properties make it difficult for researchers to synthetize a considerable number of them [182].

## 4. Pharmaceutical Applications of Marine Toxins

Assuming their pivotal role in the neuronal excitability and their high expression in pain-mediating sensory neurons, NaV channels are considered therapeutical targets in the context of acute and inflammatory pain [183]. Despite the availability of a wide variety of analgesic drugs, clinicians face a difficulty in managing pain that is resistant to standard treatments. Neurotoxins have been proven to possess an analgesic potential via blocking ion channels such as VGSC that play an important role in pain transmission. For this reason, scientists aimed to develop medicinal products using neurotoxins as pain killers. Additionally, several patents have been issued worldwide regarding the use of marine neurotoxins for the treatment of pain in humans. One method for inducing local analgesia in a mammal with pain in an epithelial tissue region consists of topically applying an appropriate amount of a long-acting NaV inhibitor, such as TTX or STX, to the affected area in a suitable pharmaceutical vehicle [184]. Systemic administration of TTX or STX in mammals experiencing pain induces analgesia [185]. Moreover, another strategy has been developed to target the neuropathic pain, a major public health issue. This technique came with the discovery that TTX has a high potency in treating central-nervous-derived neuropathic pain. It consists of using TTX, or STX, or their analogs with their tolerable physiological salts as a therapeutic product in patients suffering from central nervously derived neuropathic pain [186]. In addition, another method has combined TTX and opioid antagonists. It has been found that this combination has a surprising significant anti-pain effect, particularly in neuropathic pain, with an unanticipated overadditive impact [187].

For most patients with cancer, chemotherapy is one of the most used therapies to treat carcinomas. Chemotherapy is known to cause neuropathic pain, which in many cases leads to the failure of therapy. Moreover, allodynia and hyperalgesia can occur in a high percentage of patients. As a result, a drug has been developed for the treatment of neuropathic pain resulting from chemotherapy. The developed drug consists of VGSC blockers such as TTX or STX or their derivatives along with their adequate salts. The routes of administration of this drug can be a patch through skin, orally, subcutaneous injection, intramuscular injection, intravenous injection, etc. [188]. This same composition used in this drug was effective in treating preterm labor and/or premature birth, given that TTX effectively prevents uterine contractions [189]. Moreover, a method has been established for controlling musculoskeletal pain. The active compounds in this method are TTX or its derivatives and analogues as well as STX or its derivatives and analogues that can be given as tablets, patches, or by needles [190]. Interestingly, a TTX mask has been developed. This mask exerts many effects such as easing pain and relieving itching. The mask includes active medicinal ingredients such as TTX, citric acid as a complex solubilizer, lactose as a stabilizer, mint essential oil, zinc oxide, Vaseline, and rosin [191]. 

As previously mentioned, VGSCs are implicated in the pain signaling pathway. NaV1.7 loss-of-function mutations are responsible for a human congenital insensitivity to acute and chronic pain. Therefore, to inhibit NaV1.7, 11,13-modified STX can be used as analgesic [192]. Another compound that has been developed to treat pain conditions in mammals is 10,11-modified STX [193]. The development of a special combination of NeoSTX, bupivacaine, and epinephrine has considerably prolonged the duration of full blockage to a mechanical stimulus [194]. On another note, TTX and STX are not only used in treating pain. A previous strategy was developed with the aim to be both safe and effective. This strategy is based on a formulation consisting of TTX, lidocaine hydrochloride, cosolvents, freeze-drying excipients, and stabilizing agents [195]. On the other hand, TTX, STX, and their analogues and derivatives are used to treat nicotine dependency. This technique is based on the capacity of TTX and STX to support the de-addiction mechanism [196]. 

Aside from TTX and STX, conotoxins have proven that they can be used as therapeutic agents. A recent strategy highlighted the possible use of conotoxins peptides in the medical field. Researchers have developed a μ-conotoxin peptide essentially comprising the following amino acid sequence: Xaa1-Xaa2-Cys Cys-Xaa3-Xaa-4-Xaa5-Xaaé-Xaa1-Cys-Xaa8-Xaa9-Xaa10-Xaa11-Cys-Xaa12-Xaa13-Xaa14-Xaa15-Xaa16-Cys-Cys-Xaa17, which constitutes a bioactive fragment that can be used in pharmaceutical composition for the treatment or prevention of diseases associated with VGSC [197]. 

## 5. Conclusions

The marine ecosystem is a complex of a wide variety of organisms that are a source of active toxins interacting with voltage-gated sodium channels (VGSCs). As previously described TTX, STX, and conotoxins play an important role as blockers of VGSC. Thus, they are endowed with analgesic properties and are the subject of research in the therapeutic field as drugs to manage pain. However, these neurotoxins must be administrated at very low doses and their toxicity must be well studied for the further development of safe and effective drugs.

## Figures and Tables

**Table 1:** Other toxins from shellfish that target VGSCs.

Toxin	Source/Chemical Formula	Mechanism	References
Kalkitoxin	*Lyngbya majuscule*/C_21_H_38_N_2_OS	VGSCs blocker (including TTX-S VGSC)	[59,97,98]
Jamaicamides A	*Lyngbya majuscule* (strain JHB)/C_27_H_37_O_4_N_2_ClBr	VGSCs blocker	[59,99]
Jamaicamides B	*Lyngby majuscule* (strain JHB)/C_27_H_37_O_4_N_2_Cl	VGSCs blocker	[59,99]
Jamaicamides C	*Lyngby majuscule* (strain JHB)/C_27_H_39_O_4_N_2_Cl	VGSCs blocker	[59,99]
Crossbynols A	*Leptolyngbya crossbyana/*C_30_H_15_^79^Br_7_O_6_	VGSCs activator	[100]
Hoiamides A	*Lyngbya majuscula* and *Phormidium gracile*/C_44_H_71_N_5_O_10_S_3_	VGSCs activator at site 2	[59,101]
Hoiamides B	Marine cyanobacteria in Papua New Guinea/C_45_H_73_N_5_O_10_S_3_	VGSCs activator at site 2	[59,102]
Palmyrolide A	*Leptolyngbya* cf and *Oscillatoria* spp./C_20_H_36_O_3_N	VGSCs blocker	[103]
Palmyramide A	Cyanobacteria/C_36_H_53_N_3_O_9_	VGSC blocker	[104]

**Table 2:** Other toxins from sea anemones that target VGSCs.

Toxin		Source	References
Type I	ATX II and II	*Anemonia viridis (Anemonia sulcata)*	[112]
	ApA and ApB	*Anthopleura xanthogrammica*	[127,128]
	Ae I	*Actinia equina*	[129]
	Cp I and II	*Condylactis passiflora*	[130]
	Rc I	*Radianthus (Heteractis) crispus*	[131]
	AFT I and II	*Anthopleura fuscoviridis*	[132]
	Bc III	*Bunodosoma caissarum*	[133]
	Bg II and III	*Bunodosoma granulifera*	[134]
	Halcurin	*Halcurias* sp.	[135]
	AETX I	*Anemonia erythraea*	[136]
	Hk2	*Anthopleura* sp.	[137]
	ATX Ia and Ib	*Anemonia sulcata*	[138]
	ATX II	*Anemonia sulcata*	[112]
	ATX V	*Anemonia sulcata*	[139]
	PCR1–2, 2–1, 2–5, 2–10, 3–6, and 3–7	*Anthopleura xanthogrammica*	[140]
	ApC	*Anthopleura elegantissima*	[141]
	APE 1 to APE 5	*Anthopleura elegantissima*	[142]
	Cangitoxin	*Bunodosoma cangicum*	[143]
	Am III	*Antheopsis maculata*	[144]
	Gigantoxin II	*Stichodactyla gigantea*	[145]
Type II	RTX I, II, III, IV and V	*Radianthus (Heteractis) macrodactylus*	[146,147,148]
	Gigantoxin III	*Stichodactyla gigantea*	[145]
	Rp II, III	*Radianthus (Heteractis) paumotensis*	[117,149]
	Sh I	*Stichodactyla helianthus*	[150]
Type III	ATXIII	*Anemonia viridis*	[112,114,151]
		*Dofleinia armata*	
		*Entacmaea quadricolor*	
	Da I and II	*Dofleinia armata*	[116]
	Er I	*Entacmaea ramsayi*	[116]
	Ea I(PaTx)	*Entacmaea actinostoloides*	[116]

## Data Availability

Not applicable.
